# 1281. An Evaluation of Tebipenem *In Vitro* Activity Against a Panel of *Pseudomonas aeruginosa* Isolates with Efflux, AmpC, and OprD Mutations

**DOI:** 10.1093/ofid/ofab466.1473

**Published:** 2021-12-04

**Authors:** Brian D VanScoy, Haley Conde, Nicole Cotroneo, Ian A Critchley, Thomas R Parr, Paul G Ambrose

**Affiliations:** 1 Institute for Clinical Pharmacodynamics, Inc., Schenectady, New York; 2 Spero Therapeutics

## Abstract

**Background:**

SPR994 is an orally bioavailable pivoxil hydrobromide prodrug of tebipenem, a novel oral carbapenem with activity against Gram-positive and -negative bacteria currently under development for the treatment of urinary tract infections (UTI). *Pseudomonas aeruginosa* has become one of the most common pathogens associated with nosocomial infections. A series of susceptibility studies was designed to evaluate the activity of tebipenem against a panel of *P. aeruginosa* isolates known to have alterations in efflux pumps, outer-membrane porins, and expression of AmpC beta-lactamase enzymes compared to a subset of wild-type organisms.

**Methods:**

A panel of 20 *P. aeruginosa* isolates was subjected to tebipenem and five other challenge compounds (meropenem, ertapenem, levofloxacin, tetracycline, and carbenicillin) in order to determine minimum inhibitory concentration (MIC) values, in triplicate, using standard agar dilution methodologies. *P. aeruginosa* ATCC 27853 was used as an internal control and all reported MIC values represented the modal value.

**Results:**

Tebipenem MIC values ranged from 0.25 to >64 mg/L for the 20 isolates evaluated (**Table 1**) and were similar to those found for meropenem and ertapenem with MIC values ranging from ≤0.03 to 16 mg/L and ≤0.25 to 128 mg/L, respectively. The deletion of MexB decreased the carbapenem MIC values by 2 to 4x while deletion of MexCD-OprJ and MexXy did not. Simultaneous deficiencies in MexAB, CD, EF, and XY did not decrease MIC values in comparison with the deletion of MexAB alone. Isolates possessing a derepressed AmpC showed no significant elevation in MIC value compared to wild-type isolates while those known to have lost OprD resulted in carbapenem MIC values increasing by ~4 to 32x. The combined loss of OprD and overexpression of AmpC resulted in the highest carbapenem MIC values (≥16 mg/L). Levofloxacin, tetracycline, and carbenicillin MIC values ranged from ≤0.06 to 16 mg/L, 0.5 to 64 mg/L, and ≤0.125 to >128 mg/L, respectively. All MIC values determined for the internal control were within Clinical Laboratory Standard Institute reported values.

Table 1. Modal agar-dilution MIC values for all compounds evaluated against the 20 P. aeruginosa isolates in the challenge isolate panel

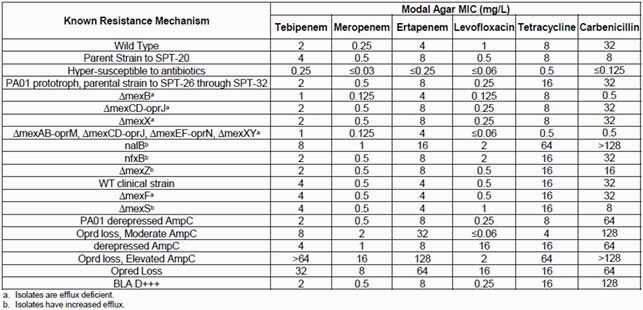

**Conclusion:**

These data provide insight into the effect that alterations in efflux pump, outer-membrane porins, and expression of AmpC enzymes have on the activity of tebipenem and comparison antibiotics.

**Disclosures:**

**Brian D. VanScoy, B.S.**, **3-V Biosciences** (Grant/Research Support)**Achogen** (Grant/Research Support)**Amplyx Pharmaceuticals, Inc.** (Grant/Research Support)**Arixa Pharmaceuticals** (Grant/Research Support)**Arsanis Inc.** (Grant/Research Support)**B. Braun Medical Inc.** (Grant/Research Support)**Basilea Pharmaceutica** (Grant/Research Support)**BLC USA** (Grant/Research Support)**Boston Pharmaceuticals** (Grant/Research Support)**Bravos Biosciences, LLC** (Grant/Research Support)**Cidara Therapeutics Inc.** (Grant/Research Support)**Cipla, USA** (Grant/Research Support)**Corcept Therapeutics** (Grant/Research Support)**Cumberland Pharmaceuticals** (Grant/Research Support)**Debiopharm International SA** (Grant/Research Support)**Discuva Limited** (Grant/Research Support)**Emerald Lake Technologies** (Grant/Research Support)**Enhanced Pharmacodynamics** (Grant/Research Support)**Entasis Therapeutics** (Grant/Research Support)**E-Scape Bio** (Grant/Research Support)**Genentech** (Grant/Research Support)**Geom Therapeutics, Inc.** (Grant/Research Support)**GlaxoSmithKline** (Grant/Research Support)**Hoffmann-La Roche** (Grant/Research Support)**Horizon Orphan LLC** (Grant/Research Support)**ICPD Biosciences, LLC** (Grant/Research Support)**Indalo Therapeutics** (Grant/Research Support)**Insmed Inc.** (Grant/Research Support)**Institute for Clinical Pharmacodynamics** (Employee)**Iterum** (Grant/Research Support)**KBP Biosciences USA** (Grant/Research Support)**Kyoto Biopharma, Inc.** (Grant/Research Support)**Matinas** (Grant/Research Support)**Meiji Seika Pharma Co., Ltd.** (Grant/Research Support)**Melinta Therapeutics** (Grant/Research Support)**Menarini Ricerche S.p.A.** (Grant/Research Support)**Merck & Co., Inc** (Grant/Research Support)**Mutabilis** (Grant/Research Support)**Nabriva Therapeutics AG** (Grant/Research Support)**Naeja-RGM Pharmaceuticals** (Grant/Research Support)**Nosopharm SAS** (Grant/Research Support)**Novartis Pharmaceuticals Corp.** (Grant/Research Support)**NuCana Biomed** (Grant/Research Support)**Paratek Pharmaceuticals, Inc.** (Grant/Research Support)**Polyphor, Ltd.** (Grant/Research Support)**Prothena Corporation** (Grant/Research Support)**PTC Therapeutics** (Grant/Research Support)**Rempex Pharmaceuticals** (Grant/Research Support)**Roche TCRC** (Grant/Research Support)**Sagimet** (Grant/Research Support)**scPharmaceuticals Inc.** (Grant/Research Support)**Scynexis** (Grant/Research Support)**Spero Therapeutics** (Grant/Research Support)**TauRx Therapeutics** (Grant/Research Support)**Tetraphase Pharmaceuticals** (Grant/Research Support)**Theravance Biopharma Pharmaceutica** (Grant/Research Support)**USCAST** (Grant/Research Support)**VenatoRx** (Grant/Research Support)**Vical Incorporated** (Grant/Research Support)**Wockhardt Bio AG** (Grant/Research Support)**Zavante Therapeutics** (Grant/Research Support)**Zogenix International** (Grant/Research Support) **Haley Conde, B.S.**, **3-V Biosciences** (Grant/Research Support)**Achogen** (Grant/Research Support)**Amplyx Pharmaceuticals, Inc.** (Grant/Research Support)**Arixa Pharmaceuticals** (Grant/Research Support)**Arsanis Inc.** (Grant/Research Support)**B. Braun Medical Inc.** (Grant/Research Support)**Basilea Pharmaceutica** (Grant/Research Support)**BLC USA** (Grant/Research Support)**Boston Pharmaceuticals** (Grant/Research Support)**Bravos Biosciences, LLC** (Grant/Research Support)**Cidara Therapeutics Inc.** (Grant/Research Support)**Cipla, USA** (Grant/Research Support)**Corcept Therapeutics** (Grant/Research Support)**Cumberland Pharmaceuticals** (Grant/Research Support)**Debiopharm International SA** (Grant/Research Support)**Discuva Limited** (Grant/Research Support)**Emerald Lake Technologies** (Grant/Research Support)**Enhanced Pharmacodynamics** (Grant/Research Support)**Entasis Therapeutics** (Grant/Research Support)**E-Scape Bio** (Grant/Research Support)**Genentech** (Grant/Research Support)**Geom Therapeutics, Inc.** (Grant/Research Support)**GlaxoSmithKline** (Grant/Research Support)**Hoffmann-La Roche** (Grant/Research Support)**Horizon Orphan LLC** (Grant/Research Support)**ICPD Biosciences, LLC** (Grant/Research Support)**Indalo Therapeutics** (Grant/Research Support)**Insmed Inc.** (Grant/Research Support)**Institute for Clinical Pharmacodynamics** (Employee)**Iterum** (Grant/Research Support)**KBP Biosciences USA** (Grant/Research Support)**Kyoto Biopharma, Inc.** (Grant/Research Support)**Matinas** (Grant/Research Support)**Meiji Seika Pharma Co., Ltd.** (Grant/Research Support)**Melinta Therapeutics** (Grant/Research Support)**Menarini Ricerche S.p.A.** (Grant/Research Support)**Merck & Co., Inc** (Grant/Research Support)**Mutabilis** (Grant/Research Support)**Nabriva Therapeutics AG** (Grant/Research Support)**Naeja-RGM Pharmaceuticals** (Grant/Research Support)**Nosopharm SAS** (Grant/Research Support)**Novartis Pharmaceuticals Corp.** (Grant/Research Support)**NuCana Biomed** (Grant/Research Support)**Paratek Pharmaceuticals, Inc.** (Grant/Research Support)**Polyphor, Ltd.** (Grant/Research Support)**Prothena Corporation** (Grant/Research Support)**PTC Therapeutics** (Grant/Research Support)**Rempex Pharmaceuticals** (Grant/Research Support)**Roche TCRC** (Grant/Research Support)**Sagimet** (Grant/Research Support)**scPharmaceuticals Inc.** (Grant/Research Support)**Scynexis** (Grant/Research Support)**Spero Therapeutics** (Grant/Research Support)**TauRx Therapeutics** (Grant/Research Support)**Tetraphase Pharmaceuticals** (Grant/Research Support)**Theravance Biopharma Pharmaceutica** (Grant/Research Support)**USCAST** (Grant/Research Support)**VenatoRx** (Grant/Research Support)**Vical Incorporated** (Grant/Research Support)**Wockhardt Bio AG** (Grant/Research Support)**Zavante Therapeutics** (Grant/Research Support)**Zogenix International** (Grant/Research Support) **Nicole Cotroneo**, **Spero Therapeutics** (Employee, Shareholder) **Ian A. Critchley, Ph.D.**, **Spero Therapeutics** (Employee, Shareholder) **Thomas R. Parr, Ph.D.**, **Spero Therapeutics** (Employee, Shareholder) **Paul G. Ambrose, Pharm.D., FIDSA**, **3-V Biosciences** (Grant/Research Support)**Achogen** (Grant/Research Support)**Amplyx Pharmaceuticals, Inc.** (Grant/Research Support)**Arixa Pharmaceuticals** (Grant/Research Support)**Arsanis Inc.** (Grant/Research Support)**B. Braun Medical Inc.** (Grant/Research Support)**Basilea Pharmaceutica** (Grant/Research Support)**BLC USA** (Grant/Research Support)**Boston Pharmaceuticals** (Grant/Research Support)**Bravos Biosciences, LLC** (Grant/Research Support, Other Financial or Material Support, member/owner)**Cidara Therapeutics Inc.** (Grant/Research Support)**Cipla, USA** (Grant/Research Support)**Corcept Therapeutics** (Grant/Research Support)**Cumberland Pharmaceuticals** (Grant/Research Support)**Debiopharm International SA** (Grant/Research Support)**Discuva Limited** (Research Grant or Support)**Emerald Lake Technologies** (Grant/Research Support)**Enhanced Pharmacodynamics** (Grant/Research Support)**Entasis Therapeutics** (Grant/Research Support)**E-Scape Bio** (Grant/Research Support)**Genentech** (Grant/Research Support)**Geom Therapeutics, Inc.** (Grant/Research Support)**GlaxoSmithKline** (Grant/Research Support)**Hoffmann-La Roche** (Grant/Research Support)**Horizon Orphan LLC** (Grant/Research Support)**ICPD Biosciences, LLC** (Grant/Research Support, Other Financial or Material Support, member/owner)**Indalo Therapeutics** (Grant/Research Support)**Insmed Inc.** (Grant/Research Support)**Institute for Clinical Pharmacodynamics** (Employee)**Iterum** (Grant/Research Support)**KBP Biosciences USA** (Grant/Research Support)**Kyoto Biopharma, Inc.** (Grant/Research Support)**Matinas** (Grant/Research Support)**Meiji Seika Pharma Co., Ltd.** (Grant/Research Support)**Melinta Therapeutics** (Grant/Research Support)**Menarini Ricerche S.p.A.** (Grant/Research Support)**Merck & Co., Inc** (Grant/Research Support)**Mutabilis** (Grant/Research Support)**Nabriva Therapeutics AG** (Grant/Research Support)**Naeja-RGM Pharmaceuticals** (Grant/Research Support)**Nosopharm SAS** (Grant/Research Support)**Novartis Pharmaceuticals Corp.** (Grant/Research Support)**NuCana Biomed** (Grant/Research Support)**Paratek Pharmaceuticals, Inc.** (Grant/Research Support)**Polyphor, Ltd.** (Grant/Research Support)**Prothena Corporation** (Grant/Research Support)**PTC Therapeutics** (Grant/Research Support)**Rempex Pharmaceuticals** (Grant/Research Support)**Roche TCRC** (Grant/Research Support)**Sagimet** (Grant/Research Support)**scPharmaceuticals Inc.** (Grant/Research Support)**Scynexis** (Grant/Research Support)**Spero Therapeutics** (Grant/Research Support)**TauRx Therapeutics** (Grant/Research Support)**Tetraphase Pharmaceuticals** (Grant/Research Support)**Theravance Biopharma Pharmaceutica** (Grant/Research Support)**USCAST** (Grant/Research Support)**VenatoRx** (Grant/Research Support)**Vical Incorporated** (Grant/Research Support)**Wockhardt Bio AG** (Grant/Research Support)**Zavante Therapeutics** (Grant/Research Support)**Zogenix International** (Grant/Research Support)

